# Survival analysis under imperfect record linkage using historic census data

**DOI:** 10.1186/s12874-024-02194-6

**Published:** 2024-03-13

**Authors:** Arielle K. Marks-Anglin, Frances K. Barg, Michelle Ross, Douglas J. Wiebe, Wei-Ting Hwang

**Affiliations:** 1 Department of Biostatistics, Epidemiology & Informatics, Perelman School of Medicine, University of Pennsylvania, Philadelphia, PA USA; 2 Department of Family Medicine and Community Health, Perelman School of Medicine, University of Pennsylvania, Philadelphia, PA USA; 3 423 Guardian Drive, Blockley Hall Room 610, Philadelphia, PA 19064 USA

**Keywords:** Census data, Censoring, Missing data, Record linkage, Survival analysis

## Abstract

**Background:**

Advancements in linking publicly available census records with vital and administrative records have enabled novel investigations in epidemiology and social history. However, in the absence of unique identifiers, the linkage of the records may be uncertain or only be successful for a subset of the census cohort, resulting in missing data. For survival analysis, differential ascertainment of event times can impact inference on risk associations and median survival.

**Methods:**

We modify some existing approaches that are commonly used to handle missing survival times to accommodate this imperfect linkage situation including complete case analysis, censoring, weighting, and several multiple imputation methods. We then conduct simulation studies to compare the performance of the proposed approaches in estimating the associations of a risk factor or exposure in terms of hazard ratio (HR) and median survival times in the presence of missing survival times. The effects of different missing data mechanisms and exposure-survival associations on their performance are also explored. The approaches are applied to a historic cohort of residents in Ambler, PA, established using the 1930 US census, from which only 2,440 out of 4,514 individuals (54%) had death records retrievable from publicly available data sources and death certificates. Using this cohort, we examine the effects of occupational and paraoccupational asbestos exposure on survival and disparities in mortality by race and gender.

**Results:**

We show that imputation based on conditional survival results in less bias and greater efficiency relative to a complete case analysis when estimating log-hazard ratios and median survival times. When the approaches are applied to the Ambler cohort, we find a significant association between occupational exposure and mortality, particularly among black individuals and males, but not between paraoccupational exposure and mortality.

**Discussion:**

This investigation illustrates the strengths and weaknesses of different imputation methods for missing survival times due to imperfect linkage of the administrative or registry data. The performance of the methods may depend on the missingness process as well as the parameter being estimated and models of interest, and such factors should be considered when choosing the methods to address the missing event times.

**Supplementary Information:**

The online version contains supplementary material available at 10.1186/s12874-024-02194-6.

## Introduction

Publicly available individual U.S. census records spanning 150 years (1790–1940), which are re-identified 72 years after the respective census dates, offer a rich resource for studying demographic, social, and economic characteristics of the U.S. population at various points in history, as well as changes over time. Census records are particularly useful for investigating sociological and epidemiological questions when matched with vital records such as birth, death, and marriage certificates from state-run registries or other data sources [1]. For example, Beach et al. [2] studied the effect of childhood typhoid exposure in the late 1800s on earnings and educational attainment later in life, by linking city-year level typhoid fatality rates to children in the 1900 census, which are then linked with adult records from the 1940 census. In another study, Ferrie et al. [3] investigated the impact of lead exposure on test scores by using the 1930 census to estimate lead exposure for children through water supplies and linking it with test scores for World War II enlistees.

However, in the absence of unique identifiers across data sources, the linkage between census records and vital records is not always successful, resulting in missing or misclassified data for a substantial portion of the census population. Unlike the decennial census which is conducted on a national level, vital registries are decentralized and managed on a state-by-state basis. They were developed much later and had uneven and sparse coverage compared to the national census, especially before 1933 [4, 5]. Federal agencies such as the National Center for Health Statistics (NCHS) were later established to collect information from the state registries in a centralized database, but coverage may not extend to the earliest years of record collection. For example, the earliest records in the National Death Index (NDI) date to 1979, whereas vital records were kept as early as 1881 in states like New York and Pennsylvania. Furthermore, the NDI uses a computerized probabilistic scoring algorithm to match vital records based on variables such as social security number, month, day, and year of birth, first and last name, and state of residence, among others. The absence or misclassification of any of these variables (for example due to changes in name or place of residence) reduces the probability of a successful match. Census records contain limited information on an individual for matching, as not all of the variables needed for successful matching are collected, leading to many missing or mismatched records.

This poses particular challenges for time-to-event analyses using historical census data linked with administrative death records. First, the event time may not be observed for some subjects. As a retrospective analysis, it is unknown whether the unobserved event times are due to a failed linkage with a vital record, or the individual being alive at the time of analysis. Second, the linkage process itself is prone to error and may result in multiple matches and false matches, particularly if the linkage variables available are insufficient for uniquely identifying an individual. Many methods exist for handling the former issue of missing data in survival analysis, and a handful are equipped for addressing the second challenge, but to our knowledge methods have not been developed for addressing both simultaneously.

Methods for handling missing survival times assume a censoring framework for the missing events. With right-censoring, the individual is lost to follow up before the event has occurred. The presence of censoring in time-to-event data is often dealt with by including censored individuals in the likelihood estimation procedure up until the time at which they are lost to follow-up. Such an approach is used in nonparametric Kaplan–Meier estimators, semi-parametric Cox proportional hazards regression, and parametric survival models such as the accelerated failure-time (AFT) model. However, in our context, the census date is the only point of observed data collection for each individual and one that is arbitrarily assigned relative to each person’s timeline. Thus, right-censoring on this date may offer little additional information compared to limiting analysis to only completely observed records.

Missing event times using historical data may also be treated as interval-censored, where the event is known to have occurred between two observed time points for an individual. Methods for this setting include cruder approaches such as imputing the event time at the beginning, midpoint or end of the interval [6]. However, this can lead to biased inference [7], particularly if the interval is large. Multiple imputation methods which make use of the information contained in the observed data are also used for interval-censoring [8–10]. However, these methods are not readily applicable to our setting of survival analysis where the lifetime of an individual is of interest, as determined using census data linked with death records. While we may be willing to assume that all individuals have died at the time of the analysis (for example, if the census occurred 100 years prior to the date of analysis), this is a large time interval between the time of the census and analysis time for using interval censoring methods. Furthermore, the aforementioned methods for interval censoring require that the upper bound for the interval is fixed and known for each individual. In our setting, the upper interval must be determined ad hoc (for example, a fixed number of years post-census, or the date of analysis). Finally, for some of the proposed methods, the imputation is iterative when fitting Cox or failure-time models, and do not readily extend to studies where there is interest in estimating the median survival time. On the other hand, for older individuals, simply right-censoring at the date of census is a very conservative approach, as enough time may have elapsed that the event has certainly occurred before the date of analysis. Novel approaches are needed to handle this unique framework using historic census records.

Methods for analyzing linked data should also account for uncertainty in the matching process, namely the potential for false or equivocal matches among the observed records. Failure to do so can lead to an underestimation of the variance and/or bias in model estimates [11, 12]. Note that we limit the scope of this work to random errors in observed matches, meaning the probability of a true linkage is independent of the linkage variables. Thus, we assume that failure to account for false matches impacts only the uncertainty around our estimates.

In this report, we seek to compare methods for handling missing event times in survival analysis using linked historical census data. We explore the performance of right-censoring (on the census date), inverse-probability weighting of the complete data, and two multiple imputation methods for estimating both median survival and the association parameters in proportional hazards and failure-time models. We are particularly interested in the repurposing of restricted mean survival and conditional survival for multiple imputation of missing event times. To account for the uncertainty in the merging process, we incorporate probabilistic scores provided by the vital record agency in our analysis. We apply the methods to study the effect of occupational and non-occupational asbestos exposure on life expectancy in a historical cohort from Ambler, PA, based on the 1930 census.

Ambler, PA was home to the nation’s largest asbestos manufacturing plant from the early 1900s to the mid-1980s. Many residents in Ambler experienced daily exposure to large amounts of asbestos in the factory as well as in their neighborhood and inside their homes. Although the asbestos factory has been closed since 1988, disposal of asbestos-containing waste continued through the majority of the twentieth century, forming several large mounds containing over 1.5 million cubic yards of asbestos waste spread over 25 acres [13]. This led to possible continuous community-level asbestos exposure through wind and water distribution channels for many years after. Several studies [14–16] have shown a clear link between exposure to asbestos and debilitating, often life-threatening, diseases such as pulmonary fibrosis, lung cancer, and mesothelioma. While the effects of exposure on mortality due to asbestos-related diseases (ARDs) have largely been studied in occupational settings, less is known about mortality among non-occupationally and environmentally exposed individuals. In this historical cohort study, census data were linked with death records obtained through matching with Ancestry.com and the National Death Index (NDI), however, there was substantial ascertainment bias in identifying death records thus motivating this work [17].

In the next section, we describe the time-to-event setting using historical census data with missing event times, followed by the proposed methods to impute the missing data. Then we perform a simulation study of the methods described, comparing them to a gold standard analysis where the outcomes are fully observed, as well as a complete case only data analysis. We then apply the methods to characterize asbestos-associated mortality in a historical cohort from Ambler, PA, and conclude with a discussion of the results.

## Methods

### Setting

We consider data where the outcome of interest is a time-to-event variable, $${T}_{i}$$. Let $${X}_{i}$$ represent a binary exposure variable of interest, and $${Z}_{i}$$ represent a covariate, where $$i=1,\dots ,n$$ indexes the $$n$$ individuals in the census cohort. In keeping with the format of historical census data, the time variable $$t\in (0,{T}_{i})$$ is defined on the scale of years since birth, and $${T}_{i}$$ represents the lifetime of an individual. For each individual, one observation time, $${W}_{i}$$, occurs corresponding with the date of the census, such that $${W}_{i}<{T}_{i}$$ for all $$i$$. We also denote the time of analysis (end of study) as $${V}_{i}$$, which (like $${T}_{i})$$ is defined using time since birth. Although the census and analysis dates are fixed calendar dates, such that $${V}_{i}-{W}_{i}=c$$ (a constant) for everyone, because our timescale is age starting at birth, $${W}_{i}$$ and $${V}_{i}$$ are specific to each individual. The true event indicator is denoted $${\delta }_{i}=I\left({T}_{i}<{V}_{i}\right)$$.

**Fig. 1:**
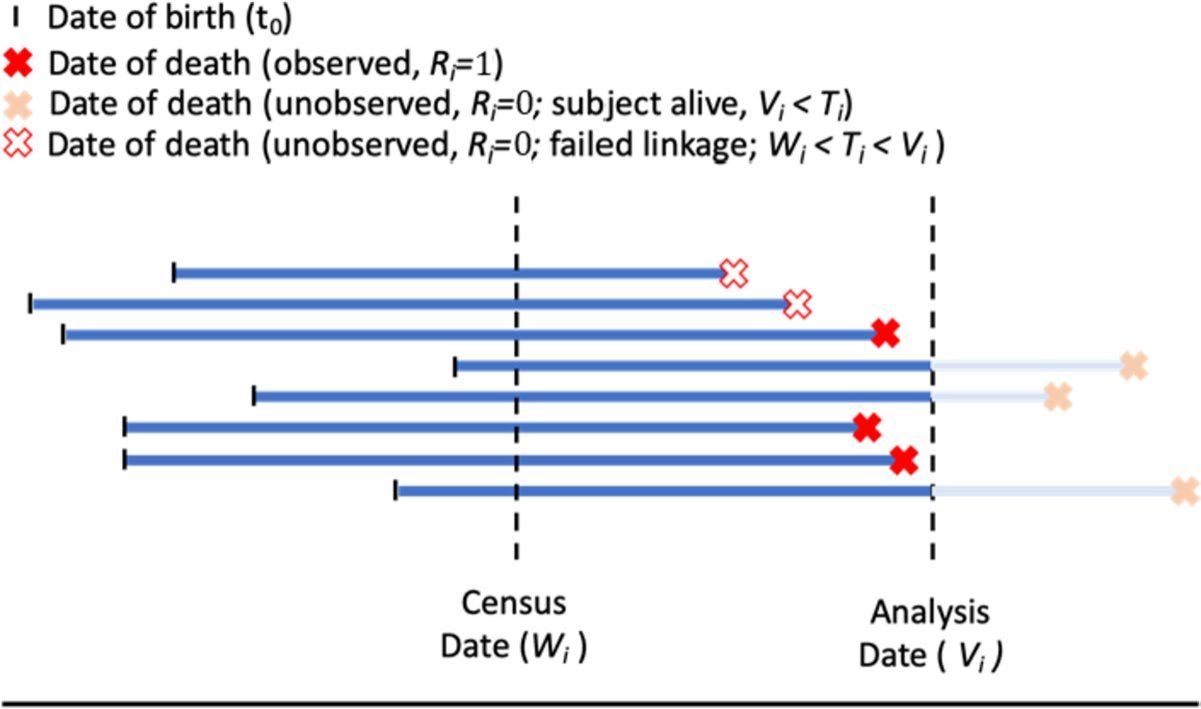
Survival framework for analysis of lifetime data using census information

Following the framework of Goldstein et al. [11], we have a primary data file, known as the file of interest (FOI), that contains linkage variables, exposure ${X}_{i}$ and the covariate of interest ${Z}_{i}$. We also have a secondary linkage data file (LDF), which contains linkage variables and an event time (which may or may not be the true event time) for those who are matched. Ideally, if linkage with all death records were successful, we would observe all event times ${T}_{i}<{V}_{i}$ in the LDF, and right-censor those who were not matched with records in the LDF at time ${V}_{i}$. However, in our setting, there is imperfect linkage. We, therefore, introduce a matching indicator, ${R}_{i}\in \left\{\mathrm{0,1}\right\},$ where ${R}_{i}=1$ if record $i$ from the FOI is matched to a record in the LDF, and ${R}_{i}=0$ if there is no match. To the investigator, it is unknown whether ${R}_{i}=0$ is due to failed linkage with a death record (i.e., if in fact ${\delta }_{i}=1$ but no match was found) or because the event has not yet occurred (${\delta }_{i}=0).$ This is illustrated more clearly in Fig. 1 below.

Furthermore, there is uncertainty in the linkage process, as the wrong record in the LDF file may be selected as a match for the FOI record. We denote the event time in the matched LDF record as ${T}_{i}^{*}$, which may or may not be equal to ${T}_{i}$. We therefore distinguish between unequivocal matches, in which there is a high probability that ${T}_{i}^{*}={T}_{i}$, and equivocal matches, where the equality is uncertain. Often, matched records from an LDF are accompanied by a probabilistic score, representing the probability that a record matched with $i$ is a true match, denoted as ${p}_{i,match}={\text{Pr}}\left({T}_{i}^{*}\in ({T}_{i}-\epsilon , {T}_{i}+\epsilon )\right)$. This probability ranges between 0 and 1. Thus, the data vector we observe for each individual is either $\{{X}_{i}, {Z}_{i}, {W}_{i},{T}_{i}^{*}, {\delta }_{i}=1,{R}_{i}=1, {p}_{i,match}\}$ or $\left\{{X}_{i}, {Z}_{i}, {W}_{i},{R}_{i}=0, {p}_{i,match}=0\right\}$.

We make two key assumptions within this framework. First, we assume that no one alive at the time of analysis (that is, ${T}_{i}\ge {V}_{i})$ is matched with a record in the LDF. Second, we assume that only one match is observed for any individual, corresponding to the record with the highest probabilistic score.

### Models and parameters of interest

Our primary interest is the estimation of the following quantities: First, the median survival time, defined as the value of *t *for which *S* (*t*) ≤ 0.5; Under perfect record linkage, it is estimated as the earliest time at which the Kaplan–Meier curve, a nonparametric estimator of survival distribution over time, falls at or below 50% survival. We seek to estimate median survival within exposure-group ${X}_{i}=\{\mathrm{0,1}\}$, denoted by ${M}_{X}$, and covariate-specific median survival times within subgroups defined by ${Z}_{i}=0$ and ${Z}_{i}=1$, denoted by ${M}_{ZX}$. Thus, we have ${M}_{x}$ =${\text{min}}\left(t\right): {S}_{KM}\left(t|{X}_{i}=x\right)\le 0.5$, and ${M}_{xz}=$ ${\text{min}}\left(t\right): {S}_{KM}\left(t|{X}_{i}=x,{Z}_{i}=z\right)\le 0.5$. Secondly, we are interested in estimating the parameters of association between ${X}_{i}$ and ${T}_{i}$ when adjusting for ${Z}_{i}$, including: (1) the log-hazard ratio for exposure ${X}_{i}$, represented by ${\beta }_{1}$ in the Cox proportional hazards (Cox PH) model,

$$\lambda \left(t\right)= {\lambda }_{0}\left(t\right){\text{exp}}\left({\beta }_{1}{X}_{i} + {\beta }_{2}{Z}_{i}\right),$$

where no parametric form is assumed for the baseline hazard, ${\lambda }_{0}\left(t\right)$, and (2) the log event-time ratio for ${X}_{i}$, represented by ${\alpha }_{1}$ in the accelerated failure-time (AFT) model,

1 $${\text{log}}\left(t\right)= {\alpha }_{0}+ {\alpha }_{1}{X}_{i}+ {\alpha }_{2}{Z}_{i}+\frac{1}{p}\epsilon ,$$

where $\epsilon$ follows an extreme value distribution (i.e. $f\left(\epsilon \right)={\text{exp}}(\epsilon -{\text{exp}}\left(\epsilon \right))$, and it is assumed that.

2 $${T}_{ij}|{X}_{i},{Z}_{i} \sim {\text{Weibull}}\left(\gamma ,p\right).$$

In Eqs. 1 and 2 above, $$p$$ is a shape parameter, and $$\gamma ={{\text{exp}}\left(-\left({\alpha }_{0}+ {\alpha }_{1}{X}_{i}+ {\alpha }_{2}{Z}_{i}\right)\right)}^{p}$$ is the scale parameter. $${\alpha }_{1}$$ can be interpreted as the log event-time ratio for being in the exposed group ($${X}_{i}=1)$$ compared to the unexposed group ($${X}_{i}=0$$). Note that under the Weibull distribution, $${\alpha }_{1}$$ in the AFT model has a direct relationship to $${\beta }_{1}$$, the log-hazard ratio from the proportional hazards model:

3 $${\beta }_{1}= -{\alpha }_{1}*p.$$

### Missing data methods

We compare the performance of various methods for estimating ${M}_{0},{M}_{1}, {M}_{00}, {M}_{01}, {M}_{10}, {M}_{11}$, ${\beta }_{1}$ and ${\alpha }_{1}$ in the presence of missing event times due to imperfect linkage, assuming missing-at-random (MAR) and missing-completely-at-random (MCAR) mechanisms, where $P\left({R}_{i}=0|{X}_{i},{Z}_{i},{T}_{i}\right)=P\left({R}_{i}=0|{X}_{i},{Z}_{i}\right)$ or $\left({R}_{i}=0|{X}_{i},{Z}_{i},{T}_{i}\right)=P({R}_{i}=0)$ respectively. We also seek to account for the uncertainty associated with equivocal matches. Note, we do not address the case where missingness depends on unobserved data, which may include the missing event times, as this would require additional assumptions on the missing-not-at-random (MNAR) process, which is outside of the scope of this paper. The methods we consider in the current report are divided into non-imputation and imputation-based methods. The non-imputation approaches include weighted and unweighted complete-case analysis. For imputation methods, we investigate multiple imputations based on the restricted-mean (MIRM) function of the survival time and the conditional survival function (MICS). We describe each of the approaches below.

### Complete-case and IPW

A complete-case approach involves restricting the analysis to individuals for whom ${T}_{i}$ is observed (${R}_{i}=0$) and the match is unequivocal (i.e., ${p}_{i,match}\ge P\in [\mathrm{0,1}]$, where $P$ is a chosen threshold for the certainty of the match). In the MCAR setting, we expect a complete-case analysis to yield unbiased, but inefficient estimates for ${\widehat{\beta }}_{1}$, while in the MAR case, a complete-case approach may result in bias.

### Inverse probability weighting (IPW)

We can extend the complete case approach to include individuals who have both unequivocal and equivocal matches (i.e. ${p}_{i,match}<P\in [\mathrm{0,1}]$). A weighted analysis is then performed, where the contribution of each observation to the estimator is weighted by the inverse of the estimated propensity for missingness, $Pr{\left({R}_{i}=0|{X}_{i},{Z}_{i}\right)}^{-1}$, as well as the probabilistic score, ${p}_{i,match}$. The weights take the following form:

4 $$\frac{1}{P\left({R}_{i}=0|{X}_{i},{Z}_{i}\right)}*{p}_{i,match}.$$

The above weights account for both the MAR and MCAR process that determine if a match is observed, and the uncertainty associated with a potential mismatch. Little and Rubin [18] showed that IPW would lead to unbiased estimates of $${\widehat{\beta }}_{1}$$ in the case of MAR. For this and the complete case approach, we do not consider censoring, as the data points are limited to those with observed matches/event times ($${R}_{i}=1)$$.

### *Censoring at*${W}_{i}$

One way to make use of the full dataset, including true matches, equivocal matches, and non-matches, is to right-censor all unmatched individuals (that is, those with unobserved death times, or ${R}_{i}=0$) at their last observed follow-up during the study, which is, in this case, the census date, ${W}_{i}$. The validity of this approach requires that censoring be unrelated to the failure time, ${T}_{i}$ (i.e. non-informative censoring) [19]. Since ${W}_{i}$ occurs on a fixed date, irrespective of ${T}_{i}$ or any characteristics of the individuals, this assumption is reasonable.

### Multiple imputation methods

Imputation is another means of including all data points in the analysis, using imputed survival times in place of the missing survival times. In a multiple-imputation procedure, multiple (we denote this number as $B$) datasets are created by imputing the missing event times $B$ times, according to an assumed model for the missing values. With the imputed data, we obtain $B$ estimates of median survival and log-HR, which are combined using Rubin’s rules [20].

In our framework of imperfect linkage, we impute event times both for individuals with no match, as well as those with equivocal matches (i.e., those with a probabilistic score, ${p}_{i,match}<P\in [\mathrm{0,1}]$). Once the event times have been imputed, model estimation proceeds using both the observed and imputed data. This means that individuals who were matched equivocally (${p}_{i,match}\le P)$ appear twice in the analytic data set: once using the matched event time, and another using the imputed event time. The matched event time will receive a weight of ${p}_{i,match}$ in model estimation, while the imputed event time receives a weight of $(1-{p}_{i,match})$. Individuals who were not matched (missing an event time) will contribute only their imputed event time to the likelihood with a weight of 1.

We investigate two multiple-imputation models for the missing and equivocal survival times: multiple imputation of the restricted mean (MIRM) and multiple imputation of conditional survival (MICS).

Recall, the restricted mean survival time (RMST) is the expected or mean value of min $\left({T}_{i},\tau \right),$ where $\tau$ is a pre-specified time limit of interest. RMST is represented as the area under the survival curve up to time $\tau ,$

5 $$E[{\text{min}}({T}_{i},\tau )]={\int }_{0}^{\tau }S\left(t\right)dt$$

Equation 5 can be thought of as the average life expectancy over a fixed time interval, $(0,\tau )$, as opposed to a more general interpretation of mean survival that does not account for temporal differences in event-time distribution [21]. Imputing mean survival restricted to $\tau$ is of interest in our study context, as we would not expect persons to live beyond a certain age, for example, 100 years. Furthermore, Liu, Murray, and Tsodikov [22] introduced an algorithm for imputing RMST as a function of covariates. The algorithm first fits a modified AFT model to the complete observations (those with ${R}_{i}=0$) that accounts for the restricted mean structure, as follows

6 $$E\left[{\text{log}}\left({\text{min}}\left({T}_{i},\tau \right)\right)\right]= {\alpha }_{0}+ {\alpha }_{1}{X}_{i}+ {\alpha }_{2}{Z}_{i}$$

With the imputation proceeds on the scale of ${\text{log}}\left({\text{min}}\left({T}_{i},\tau \right)\right)$. For each of the imputed datasets, ${\left\{{\text{log}}\left({\text{min}}\left({T}_{i},\tau \right)\right)\right\}}_{i=1}^{{n}_{k}}$ are generated from a multivariate normal distribution with mean equal to the fitted values from RMST model and the corresponding covariance matrix.

For MICS approach, we recall that conditional survival is defined as the probability of surviving a further $u$ years, having survived up to time $t$. This is different from overall survival, which refers to the probability of surviving to $t$ years from time 0. Conditional survival, denoted as ${S}_{C}\left(u+t|t\right)$, is evaluated as

$${S}_{C}\left(u+t|t\right)=\frac{S\left(u+t\right)}{S(t)}$$

In the context of missing data, this distribution is useful for imputing event times conditional on surviving to time *t* [23]. We seek to impute using its related cumulative distribution function (CDF),

7 $${F}_{c}\left(u+t|t\right)=1-{S}_{c}(u+t|t)$$

Since all study participants were observed at the date of the census, we could impute the missing death times conditional on having survived to time ${W}_{i}$. We estimate conditional survival probabilities using the observed data under a Weibull AFT working model. Specifically, $S\left({T}_{i}|{X}_{i},{Z}_{i}\right)={\text{exp}}(-{\gamma }_{i}{T}_{i}^{p})$, where ${\gamma }_{i}={{\text{exp}}\left(-\left({\widehat{\alpha }}_{0}+ {\widehat{\alpha }}_{1}{X}_{i}+ {\widehat{\alpha }}_{2}{Z}_{i}\right)\right)}^{p}$. Then

8 $$F\left({u}_{i}+{W}_{i}|{W}_{i},{X}_{i}, {Z}_{i}\right)=1-{\widehat{S}}_{C}\left({u}_{i}+{W}_{i}|{W}_{i}, {X}_{i}, {Z}_{i}\right)=1-\frac{\widehat{S}\left({u}_{i}+{W}_{i}|{X}_{i},{Z}_{i}\right)}{\widehat{S}\left({W}_{i}|{X}_{i},{Z}_{i}\right)}=1- \frac{{\text{exp}}\left(-{\upgamma }_{{\text{i}}}\right.{\left({u}_{i}+{W}_{i}\right)}^{p})}{{\text{exp}}(-{\upgamma }_{{\text{i}}}{W}_{i}^{p})}$$

With this distribution, we can impute any percentile of the CDF using probability integral transformation. We randomly generate percentiles ${q}_{i}$ as Uniform(0,1) and impute the missing death time, calculated as ${u}_{i}+{W}_{i}$, as follows:

9 $$\begin{array}{c}F\left({u}_{i}+{W}_{i}|{W}_{i},{X}_{i}, {Z}_{i}\right)={q}_{i}=1- \frac{{\text{exp}}\left(-{\upgamma }_{{\text{i}}}\right.{\left({u}_{i}+{W}_{i}\right)}^{p})}{{\text{exp}}(-{\upgamma }_{{\text{i}}}{W}_{i}^{p})}\\ \Rightarrow {\text{log}}\left(1-{q}_{i}\right)= {\upgamma }_{{\text{i}}}{W}_{i}^{p}-{\upgamma }_{{\text{i}}}{\left({u}_{i}+{W}_{i}\right)}^{p}\\ \begin{array}{c}\Rightarrow {\left({u}_{i}+{{\text{W}}}_{{\text{i}}}\right)}^{p} = {W}_{i}^{p}-\frac{ {\text{log}}\left(1-{q}_{i} \right)}{{\upgamma }_{{\text{i}}}}\\ \Rightarrow {u}_{i}+{{\text{W}}}_{{\text{i}}} = {\left[{W}_{i}^{p}-\frac{ {\text{log}}\left(1-{q}_{i} \right)}{{\upgamma }_{{\text{i}}}}\right]}^{1/p}={T}^{imp}\end{array}\end{array}$$

The imputed event times can all be treated as observed, or we can apply the right-censoring at the time ${V}_{i}$ for those with imputed time ${T}_{i}^{imp}>{V}_{i}$, to mimic a gold-standard analysis where all ${T}_{i}\le {V}_{i}$ are observed and ${T}_{i}>{V}_{i}$ are censored. We use the latter approach in our simulations and data application.

The approaches described above are summarized in Table 1 below.

**Table 1:** Weighting schemes for the proposed missing data methods

Weights for unequivocal matches	Weights for equivocal matches	Weights for nonmatches
**Complete case (CC)**		
1	0	0
**Inverse probability weighting of all matches (IPW)**		
$\frac{1}{{\text{Pr}}\left({R}_{i}=1|{X}_{i},{Z}_{i}\right)}$	$\frac{1}{{\text{Pr}}\left({R}_{i}=1|{X}_{i},{Z}_{i}\right)}*{p}_{i,match}$	0
**Censoring nonmatches at ** ${{\varvec{W}}}_{{\varvec{i}}}$ ** (CENS)**		
1	$${p}_{i,match}$$	1 (for $${W}_{i})$$
**Multiple imputation for equivocal matches and nonmatches (MIRM and MICS)**		
1	${p}_{i,match}$(for ${T}_{i}$) $1-{p}_{i,match}$ (for ${T}_{i}^{imp}$)	1 (for ${T}_{i}^{imp}$)

### Simulation study

#### Design

We conduct a simulation study to evaluate the performance of the 5 missing data methods described (CC, IPW, CENS, MIRM, MICS) on the estimation of covariate-specific median survival (i.e. median survival within subgroups defined by $${X}_{i}$$ and $${Z}_{i}$$, denoted as ($${M}_{00},{M}_{01},{M}_{10},{M}_{11})$$), covariate-averaged median survival (median survival for $${X}_{i}=0$$ and $${X}_{i}=1$$, averaged over the distribution of the covariate $${Z}_{i}$$, denoted as $${(M}_{0}, {M}_{1})$$) and the effect parameters from the Cox PH ($${\beta }_{1}$$) and Weibull AFT ($${\alpha }_{1})$$ models. Data are simulated to reflect the historical census setting where everyone in the study population is observed at the date of the census, but event times are MCAR or MAR for a subset of individuals. The analysis date is set to occur 50 years after the census date, thus for the simulation we have $${V}_{i}-{W}_{i}=50$$ years. The performance of the missing data methods is evaluated in comparison to a gold-standard analysis, in which we observe all death times that occur before $${V}_{i}$$, and those still alive at $${V}_{i}$$ are right-censored at $${V}_{i}$$. We denote this gold-standard analysis as ‘Fully Observed’.

We consider five settings, where survival and/or missingness may depend on the exposure of interest, ${X}_{i}$, or covariate ${Z}_{i}$, or both ${X}_{i}$ and ${Z}_{i}$. If missingness depends on ${Z}_{i}$ only, while the outcome model includes ${X}_{i}$ only, then missingness is MCAR. However, if ${Z}_{i}$ is also predictive of survival, or both the survival and missingness depend on ${X}_{i}$, then missingness is MAR. Specifically, ${T}_{i}\sim {\text{Weibull}}{\left(\gamma ={\text{exp}}\left({-(\alpha }_{0}+ {\alpha }_{1}{X}_{i}+ {\alpha }_{2}{Z}_{i}\right)\right)}^{p}, p=6$), where ${X}_{i}\sim {\text{Binomial}}(0.5)$ and ${Z}_{i}\sim {\text{Binomial}}(0.5)$. The missingness indicator is generated as ${R}_{i}\sim {\text{Binomial}}({\text{exp}}\left({\delta }_{0}+ {\delta }_{1}{X}_{i}+ {\delta }_{2}{Z}_{i}\right)).$ Values for $\boldsymbol{\alpha }$ are chosen to reflect possible lifetime distributions in an association study comparing a healthy population to an exposed population. In settings where survival time and/or missingness depend on only one variable, the parameter corresponding to the excluded variable is set to 0. This is described in more detail in Table 2 below. Age at the time of the census is generated as ${W}_{i}\sim {\text{Uniform}}\left(0,{T}_{i}\right)$.

**Table 2:** Simulation study design

Setting	$1$	2	3	$4$	5
Missingness	MCAR	MAR	MAR	MAR	MAR
Data generation	${T}_{i}\sim {X}_{i},$ ${R}_{i}\sim {Z}_{i}$	${T}_{i}\sim {X}_{i}$, ${R}_{i}\sim {X}_{i}$	${T}_{i}\sim {X}_{i}+{Z}_{i}$, ${R}_{i}\sim {Z}_{i}$	${T}_{i}\sim {X}_{i}+{Z}_{i}$, ${R}_{i}\sim {X}_{i}+ {Z}_{i}$	${T}_{i}\sim {X}_{i}+{Z}_{i}$, ${R}_{i}\sim {X}_{i}+ {Z}_{i}$
${{\alpha }_{0}, \alpha }_{1}, {\alpha }_{2}$	4.4, -0.2, 0	4.4, -0.2, 0	$4.5, -0.2, -0.2$	$4.5, -0.2, -0.2$	$4.5, -0.2, -0.2$
${\delta }_{0}, {\delta }_{1}, {\delta }_{2}$	-1, 0, 2	-1, 2, 0	-1, 0, 2	-1, 1, 1	-1, 1, 1
${\beta }_{1}$(Cox PH)	1.2	1.2	1.2	1.2	1.2
${p}_{i,match}$	Beta (8,2)	Beta (8,2)	Beta (8,2)	Beta (8,2)	Beta (8 – ${Z}_{i}$, 2 + ${Z}_{i}$)
Median survival (yrs)	${M}_{1}=$ 63.3 ${M}_{0}=$ 78.0	${M}_{1}=$ 63.3 ${M}_{0}=$ 77.9	${M}_{1}=$ 62.9 ${M}_{0}=$ 77.6	${M}_{1}=$ 62.8 ${M}_{0}=$ 77.5	${M}_{1}=61.5$ ${M}_{0}=$ 77.6
Max time generated (yrs)	123.5	125.1	130.6	131.0	132.6

We further introduce some random error to the matching process in the form of measurement error, using a randomly generated probabilistic score. A probabilistic score, $${p}_{i,match}$$ is produced for all observed matches and follows a Beta (8,2) distribution. For those with $${p}_{i,match}>0.8$$ (i.e. true or unequivocal matches), we set the matched event time to be equal to their true event time (i.e. $${T}_{i}^{*}={T}_{i})$$. For those with $${p}_{i,match }<0.8$$ we introduce error to the matched event time as:

10 $${T}^{*}={T}_{i}+{\phi }_{i},$$

where ${\phi }_{i}\sim N\left(0, {\left({1.8}^{1/{p}_{i,match}}\right)}^{2}\right)$. Thus, the smaller ${p}_{i,match}$ is, the greater the measurement error. The fifth simulation setting modifies the ${p}_{i,match}$ distribution to be dependent on${Z}_{i}$, such that the likelihood of an unequivocal match is lower when${Z}_{i}=1$. This is to reflect real world settings where the quality and accuracy of linkage variables may vary based on individual characteristics (for example, name changes for married women, or a lack of available data for foreign-born individuals).

In all settings, we include both ${X}_{i}$ and ${Z}_{i}$ in the model for the censoring weights in IPW. We assume the correct specification of the final survival models by including the same variables in imputation and analysis as we use in data generation. For MIRM, values for $\tau$ (80 and 120) were selected that were (1) sufficiently different so as to show sensitivity of performance to $\tau$ and (2) were near to the median and upper bound, respectively, of the empirical distribution of survival times generated (reported in Table 2 above).

Cox PH and AFT models are fit using the *survival::coxph()* and *survival::survreg()* functions in R respectively.

In each of the four settings, we perform $K$=500 simulations. For the ${k}^{th}$ iteration, a dataset of size $n=1000$ is generated, and estimates for the parameters of interest, denoted as ${\widehat{\beta }}_{1}^{(k)},{\widehat{\alpha }}_{1}^{(k)}, {\widehat{M}}_{0}^{(k)}$, ${\widehat{M}}_{1}^{(k)},$ ${\widehat{M}}_{00}^{(k)}, {\widehat{M}}_{01}^{(k)}, {\widehat{M}}_{10}^{(k)}, {\widehat{M}}_{11}^{(k)}$ and ${\widehat{M}}_{1}^{(k)}-{\widehat{M}}_{0}^{(k)}$, are obtained using each of the following: the fully observed data (gold-standard), complete cases only (without weighting), IPW, CENS, MIRM, and MICS. Empirical mean bias is calculated for ${\widehat{\beta }}_{1},{\widehat{\alpha }}_{1}, {\widehat{M}}_{0},{\widehat{M}}_{1},{\widehat{M}}_{00},{\widehat{M}}_{01},{\widehat{M}}_{10},$ ${\widehat{M}}_{11}$ and ${\widehat{M}}_{1}-{\widehat{M}}_{0}$ overall $K$ iterations with respect to the gold-standard estimates, as well as empirical standard errors for ${\widehat{\beta }}_{1}$ and ${\widehat{\alpha }}_{1}$. Model-based standard errors for ${\widehat{\beta }}_{1}^{(k)}$ and ${\widehat{\alpha }}_{1}^{(k)}$ are obtained from the outputted covariance matrices of the *coxph* and *survreg* functions in R, respectively.

### Association between exposure and outcome

**Fig. 2:**
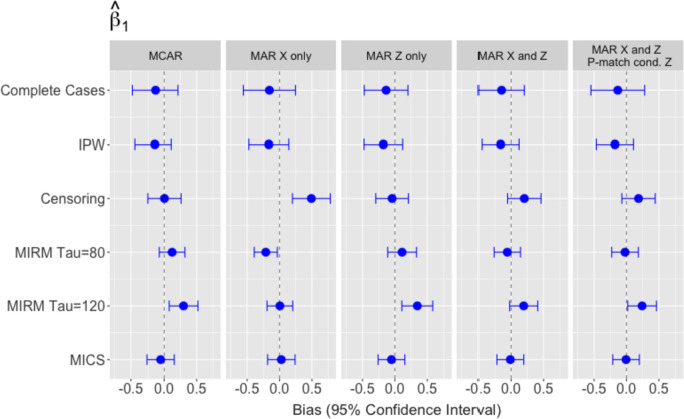
Empirical bias and model-based confidence intervals for ${\widehat{\beta }}_{1}$: (1) MCAR, (2) is MAR with both ${T}_{i}$ and ${R}_{i}$ dependent on ${X}_{i}$ only, (3) MAR with ${T}_{i}$ dependent on ${X}_{i}$ and ${Z}_{i}$, while ${R}_{i}$ depends on ${X}_{i}$ only, (4) MAR with both ${T}_{i}$ and ${R}_{i}$ dependent on ${X}_{i}$ and ${Z}_{i}$

**Fig. 3:**
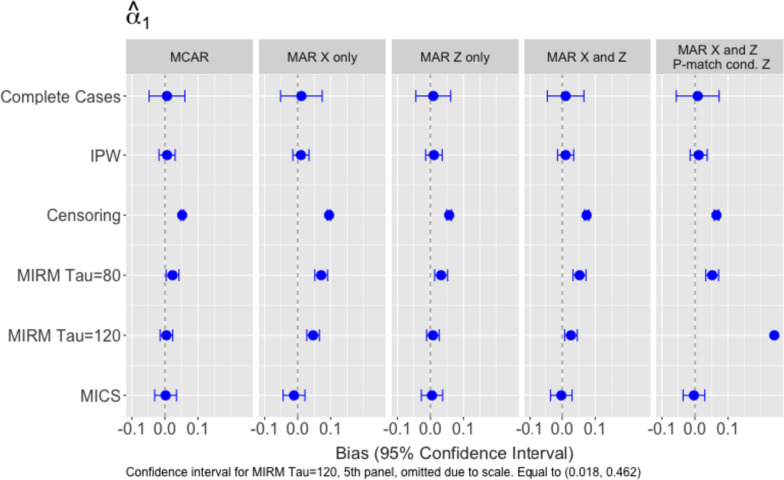
Empirical bias and model-based confidence intervals for ${\widehat{\alpha }}_{1}$: (1) MCAR, (2) is MAR with both ${T}_{i}$ and ${R}_{i}$ dependent on ${X}_{i}$ only, (3) MAR with ${T}_{i}$ dependent on ${X}_{i}$ and ${Z}_{i}$, while ${R}_{i}$ depends on ${X}_{i}$ only, (4) MAR with both ${T}_{i}$ and ${R}_{i}$ dependent on ${X}_{i}$ and ${Z}_{i}$

Simulation results for the exposure-outcome association parameters (the log hazard ratio and log event time ratio) can be found in Figs. 2 and 3, which show that the relative performance of the missing data methods varies based on the model used and the setting. When fitting a Cox PH model, both the weighted (IPW) and unweighted complete case analyses underestimate ${\beta }_{1}$ under all MCAR and MAR settings (Fig. 2) when compared to the fully observed ‘gold-standard’ analysis, as the IPW only improved efficiency. Censoring at ${W}_{i}$ produces unbiased estimates of ${\beta }_{1}$ when missingness is MCAR or MAR with dependence on covariate ${Z}_{i}$ only. However, when missingness is influenced by the exposure variable ${X}_{i}$, censoring at ${W}_{i}$ overestimates ${\beta }_{1}$. Imputing based on conditional survival (MICS) reduces bias in all four settings and produces narrower confidence intervals compared to censoring, complete case analysis or IPW. Results for MIRM vary substantially based on the value of the upper bound $\tau$, with the less restrictive bound ($\tau =1$ 20 years) yielding less biased estimates compared to $\tau =80$.

Performance of the methods when estimating ${\alpha }_{1}$ from an AFT model (Fig. 3) contrast sharply from their Cox model results. IPW and the unweighted complete case analysis produce the least biased estimates of ${\alpha }_{1}$. Furthermore, IPW improves precision in comparison to the unweighted analysis, with similar efficiency gains as MICS. MICS again produces estimates with low bias, comparable with IPW, but with wider confidence intervals. Censoring at ${W}_{i}$ leads to severe bias when estimating ${\alpha }_{1}$ in all settings. Conversely to the Cox model results, MIRM performed better with the higher bound ($\tau =120)$ compared to $\tau =80$ in all settings except for when ${p}_{i,match}$ depends on ${Z}_{i}$.

### Median survival times

**Fig. 4:**
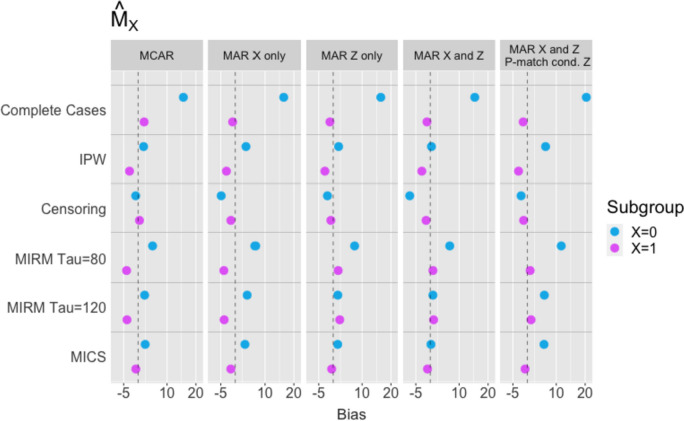
Empirical bias for ${\widehat{M}}_{0}$ and ${\widehat{M}}_{1}$: (1) MCAR, (2) MAR with both ${T}_{i}$ and ${R}_{i}$ dependent on ${X}_{i}$ only, (3) MAR with ${T}_{i}$ dependent on ${X}_{i}$ and ${Z}_{i}$, while ${R}_{i}$ depends on ${X}_{i}$ only, (4) MAR with both ${T}_{i}$ and ${R}_{i}$ dependent on ${X}_{i}$ and ${Z}_{i}$

**Fig. 5:**
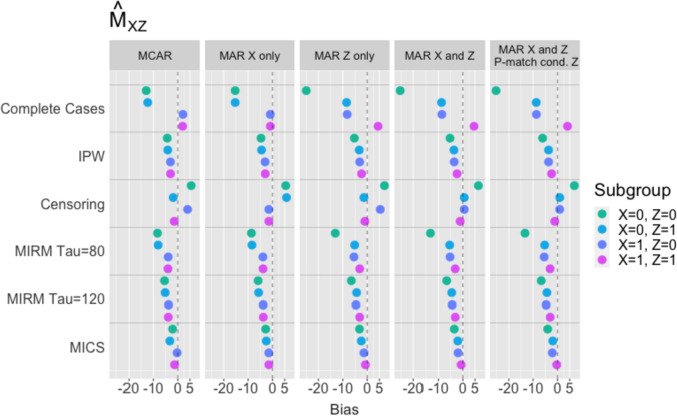
Empirical bias for ${\widehat{M}}_{00}$, ${\widehat{M}}_{01}$, ${\widehat{M}}_{10}$ and ${\widehat{M}}_{11}$: (1) MCAR, (2) MAR with both ${T}_{i}$ and ${R}_{i}$ dependent on ${X}_{i}$ only, (3) MAR with ${T}_{i}$ dependent on ${X}_{i}$ and ${Z}_{i}$, while ${R}_{i}$ depends on ${X}_{i}$ only, (4) MAR with both ${T}_{i}$ and ${R}_{i}$ dependent on ${X}_{i}$ and ${Z}_{i}$

In the simulation results for median survival times (Figs. 4 and 5), MICS most consistently results in low bias when estimating median survival within exposure groups $${X}_{i}=0$$ and $${X}_{i}=1$$, as well as covariate-dependent median survival (i.e., within subgroups defined by both $${Z}_{i}$$ and $${X}_{i})$$. This method produces estimates close to the fully observed, gold-standard approach, in all MCAR and MAR settings. It is, however, outperformed by MIRM with large $$\tau$$ when estimating $${M}_{X}$$ in MAR settings. The IPW approach also reduces bias compared to the complete case analysis but is outperformed by MICS. Censoring at $${W}_{i}$$ reduces bias in exposure-specific median survival, but results in greater bias for more disaggregated estimates. Note that regardless of method, the bias in estimating $${M}_{0}$$ increases when $${p}_{i,match}$$ is depends on $${Z}_{i}$$.

### Sensitivity analysis

A sensitivity analysis was performed to better understand the importance of imputation model specification on the performance of the multiple imputation approaches (MIRM and MICS). We used misspecified models for imputation, including one that omitted covariate ${Z}_{i},$ and one that had an interaction between ${X}_{i}$ and ${Z}_{i}$. The results (Figures A.1. – A.4 in the appendix) suggest that for all methods, the Cox-based hazard ratio as well as median survival can be biased under imputation model misspecification, while the AFT-based hazard ratio was more robust to misspecification. Greater bias was observed as a result of covariate omission as opposed to inclusion of an interaction term.

### Application to historical ambler cohort data

A historical cohort of individuals living in Ambler, PA was derived from 1930 census data. The cohort was created to study the effects of occupational, paraoccupational, and environmental asbestos exposure on life expectancy. Data on 4,514 adult residents from the 1930 census was publicly available on Ancestry.com, including individual demographic information: name, address, household identifier, household members, birth year, birthplace, race, sex, and occupation. Individuals were classified as having occupational exposure to asbestos if their listed place of work was one of the following: asbestos, shingles plant, shingle mill, chemical plant, chemical works, chemical, chemical manufacturer, mill. Paraoccupational exposure, a form of non-occupational exposure, was defined as having the same residential address as an individual with occupational exposure. For individuals without a listed house number, exposure was classified based on the listed familial relationship to the occupationally exposed individual (e.g., wife, son, daughter).

The outcome of interest was overall mortality, with survival time operationalized as the age of death. The vital status of the individuals in the cohort was first obtained through searches on Ancestry.com, which features mortality data from a variety of death-related archives, the primary of which include Pennsylvania Death Certificates, U.S. Social Security Death Index, and the U.S. Grave Index. For individuals whose death data could not be fully identified through Ancestry.com, attempts were made to match them with National Death Index (NDI) records using additional identifiers such as social security numbers. Note that the NDI only contains information on deaths from 1979 onwards. Where discrepancies in death record dates occurred, the NDI record was used if the probabilistic matching score variable (a measure of the quality of matching provided by NDI [24] exceeded 30.

To estimate the median survival time and association parameters for the occupational and para-occupational asbestos exposure on life expectancy, Kaplan Meier curves, Cox PH and Weibull AFT models were fit using a complete-case analysis, IPW, MIRM and MICS. Analysis models adjusted for age, sex, race, and place of birth (U.S. vs. non-U.S.). For inverse probability weighting, the propensity scores for missingness were modeled as a function of birthplace, race, sex, and age. Probabilistic matching scores from the NDI were transformed to the 0,1-scale. On the new scale, a score of 1 was considered an unequivocal match. Death dates identified through Ancestry.com were also treated as true matches (probabilistic score of 1).

sA total of 4507 individuals were included in the analysis with complete covariate information, in which 87.5% of individuals were of white ethnicity and 12.4% were black, 49.3% were female and 15.9% were born outside of the U.S. The average age was 29.6 years (± 20.3 years). 10.5% of individuals were occupationally exposed to asbestos, while 36.2% had para-occupational exposure. Overall, death dates were identified for 2,440 individuals (54% of the cohort). Population characteristics stratified by exposure type and event time observation are summarized in Tables 3 and 4. As observed by Wortzel et al. [17] and confirmed by our results in Table 4, ascertainment bias for death-related data exists for this cohort, as those who were U.S. born, older, male, and of white ethnicity were more likely to have their death dates identified. Being male, U.S. born, white and occupational exposure were also associated with higher probabilistic matching scores (that is, better quality matches). These groups were also less likely to be occupationally or para-occupationally exposed to asbestos (see Table 3). Assuming ascertainment was unrelated to life expectancy, we sought to implement the aforementioned methods in handling this MAR problem.

**Table 3:** Characteristics of the study population by exposure type (*n* = 4514)

	**Occupational**			**Para-Occupational**		
Variable	Exposed (*n* = 473)	Non-Exposed (*n* = 4037)	Difference	Exposed (*n* = 1635)	Non-Exposed (*n* = 2875)	Difference
Age at census (yrs)	38.5 ± 14.5	28.6 ± 20.6	9.9*	24.6 ± 18.8	32.5 ± 20.5	-7.9*
Males (%)						
	418(88%)	1867(46%)	42%*	766(47%)	1519(53%)	-6.0%*
Race (%)						
White	385(81%)	3563(88%)	-6.9%* 6.9%*	1314(80%)	2634(92%)	-11.1%* 11.1%*
Black	88(19%)	472(12%)		321(20%)	239(8%)	
Other	0 (-)	2 (-)	-	0 (-)	2 (-)	-
Non-U.S. born (%)	195(41.2%)	520(12.9%)	28.3%*	316(19.3%)	399(13.9%)	5.4%*

*Indicates statistically significant difference at the 0.05 significance level, based on Wilcoxon rank-sum test for age, and chi-square test for other variables

**Table 4:** Characteristics of the study population by missing or observed death times (*n* = 4514)

Variable	Missing (*n* = 2074)	Observed (*n* = 2440)	Difference
Age at census (yrs)	24.94 ± 19.09	33.58 ± 20.39	8.64*
Males (%)	872 (42.1%)	1413 (57.9%)	16.8%*
Non-U.S. born (%)	372 (17.9%)	347 (14.2%)	3.7%*
Race (%)			
White	1757 (84.9%)	2191 (89.8%)	4.9%* -4.8%*
Black	311 (15.0%)	249 (10.2%)	
Other	2 (0.1%)	0 (-)	0.1%
Occ Exp (%)	214 (10.3%)	259 (10.6%)	0.3%
ParaOcc Exp (%)	883 (42.7%)	752 (30.8%)	-11.8%*

* Indicates statistically significant difference at the 0.05 significance level, based on Wilcoxon rank-sum test for age, and chi-square test for other variables

Table 5 suggests that the probabilistic scores are associated with individual characteristics. Since this impacts the relative performance of MIRM with different $\tau$ (as shown in simulations) and given that median and maximum survival times among true matches are 71.77 and 109.8 years respectively, both $\tau =80$ and $\tau =110$ were used in implementing the MIRM method.

**Table 5:** Probabilistic score distribution by individual characteristics for matches

Variable	Ancestry.com and NDI matches (Ancestry.com matches are treated as true matches)		NDI matches only		
	n	Avg. probabilistic score (95% CI)	n	Avg. probabilistic score (95% CI)	% true NDI matches
Female	1027	0.92 (0.91, 0.93)	273	0.71 (0.68, 0.74)	9.2%
Male	1413	0.94 (0.94, 0.95)	453	0.83 ( 0.81, 0.84)	3.0%
U.S	2093	0.93 (0.93, 0.94)	672	0.79 (0.78, 0.81)	5.4%
Non-U.S	347	0.95 (0.93, 0.97)	54	0.67 (0.59, 0.74)	0%
White	2191	0.94 (0.93, 0.95)	651	0.79 (0.78, 0.81)	5.5%
Black	249	0.91 (0.88, 0.93)	75	0.69 (0.63, 0.74)	0%
Occ Exp	259	0.98 (0.97, 0.99)	35	0.83 (0.76, 0.9)	2.9%
Non-Occ Exp	2181	0.93 (0.92, 0.94)	691	0.78 (0.76, 0.8)	5.1%
ParaOcc Exp	752	0.91 (0.90, 0.93)	272	0.76 (0.73, 0.79)	2.6%
Non-ParaOcc Exp	1688	0.95 (0.94, 0.95)	454	0.8 (0.78, 0.82)	6.4%

Table 6 shows the median survival estimates for the overall cohort and within groups defined by occupational exposure, para-occupational exposure, race, and sex. We observe that the median survival was lower for black residents compared to white residents and for males compared to females. Overall and within groups, the median survival times were lower among individuals who were occupationally exposed or para-occupationally exposed, compared to those who were unexposed. In all groups, MIRM produced the lowest estimated median survival.

**Table 6:** Unadjusted median survival by occupational and para-occupational exposure

Method	n	Median Survival						
		Overall	Occupational Exposure			Para-Occupational Exposure		
			X = 0		X = 1	X = 0		X = 1
**All**	4507							
Complete Cases		71.77	72.33		69.90	72.55		69.51
IPW		73.60	74.00		70.92	74.28		72.11
MIRM Tau = 80		65.49	65.45		65.71	66.31		64.32
MIRM Tau = 110		66.51	66.51		66.79	67.44		65.24
MICS		70.17	70.32		69.31	70.32		67.94
**Black**	560							
Complete Cases		63.53	62.99		63.53	69.00		54.73
IPW		66.68	66.29		68.37	71.60		61.73
MIRM Tau = 80		60.89	61.08		59.94	60.89		61.97
MIRM Tau = 110		61.12	61.42		60.18	62.25		60.21
MICS		63.59	63.77		62.66	66.27		61.18
**White**	3947							
Complete Cases		72.44	72.93		80.81	72.94		71.39
IPW		74.28	74.75		71.29	74.74		73.80
MIRM Tau = 80		66.04	65.98		66.31	66.64		65.03
MIRM Tau = 110		67.22	67.18		67.38	67.89		66.10
MICS		71.01	71.16		70.14	71.72		69.58
**Male**	2282							
Complete Cases		69.52	69.09		70.05	70.39		66.29
IPW		71.44	71.66		70.92	72.09		69.92
MIRM Tau = 80		63.96	63.31		65.70	65.26		62.15
MIRM Tau = 110		64.66	63.92		66.58	66.02		62.62
MICS		68.39	68.08		69.42	69.77		65.72
**Female**	2225							
Complete Cases		74.84	75.00		61.04	75.92		72.68
IPW		75.92	75.96		70.81	76.68		74.84
MIRM Tau = 80		66.30	66.33		65.80	67.05		65.34
MIRM Tau = 110		67.72	67.73		67.19	68.65		66.54
MICS		71.94	72.06		67.63	73.20		70.01

Further analysis using semi-parametric Cox PH models (Table 7) and parametric AFT models (Table 8) revealed that the observed differences in survival by para-occupational exposure were non-significant, except for the MIRM result for black residents. A significant overall effect of occupational exposure on survival was observed using IPW under the Cox PH model. Similar results were observed among the black subpopulation and male subpopulation, with the impact of occupational exposure being most severe for black. In all subgroups, MIRM estimates deviated sharply from the other methods, though not in a consistent direction.

**Table 7:** Hazard ratio (HR) estimates from Cox PH model for occupational and para-occupational exposure, adjusting for age, sex and race. Figures in bold indicate statistically significant (*p*< 0.05) effects

Method	n	Cox Proportional Hazards Model,${\text{exp}}\left({\widehat{\beta }}_{1}\right)$				
		Occupational Exposure			Para-Occupational Exposure	
		HR	95% CI		HR	95% CI
**All**	4507					
Complete Cases		**1.18**	**(1.02, 1.36)**		1.02	(0.94, 1.12)
IPW		**1.16**	**(1.004, 1.34)**		1.01	(0.92, 1.11)
MIRM Tau = 80		1.10	(0.93, 1.31)		1.04	(0.87, 1.25)
MIRM Tau = 110		1.14	(0.94, 1.38)		1.02	(0.83, 1.26)
MICS		1.14	(0.999, 1.30)		1.02	(0.93, 1.12)
**Black**	560					
Complete Cases		**1.59**	**(1.10, 2.30)**		1.19	(0.92, 1.55)
IPW		**1.50**	**(1.06, 2.12)**		1.22	(0.94, 1.59)
MIRM Tau = 80		1.16	(0.82, 1.64)		**1.57**	**(1.16, 2.11)**
MIRM Tau = 110		1.20	(0.84, 1.71)		**1.53**	**(1.11, 2.12)**
MICS		**1.31**	**(1.002, 1.70)**		1.14	(0.94, 1.38)
**White**	3947					
Complete Cases		1.13	(0.97, 1.32)		1.00	(0.91, 1.10)
IPW		1.12	(0.96, 1.32)		0.98	(0.89, 1.09)
MIRM Tau = 80		1.11	(0.93, 1.32)		0.99	(0.83, 1.19)
MIRM Tau = 110		1.15	(0.95, 1.39)		0.97	(0.79, 1.20)
MICS		1.11	(0.96, 1.29)		1.00	(0.91, 1.11)
**Male**	2282					
Complete Cases		**1.19**	**(1.03, 1.39)**		1.06	(0.94, 1.19)
IPW		**1.17**	**(1.01, 1.36)**		1.03	(0.91, 1.16)
MIRM Tau = 80		1.04	(0.89, 1.21)		1.08	(0.90, 1.30)
MIRM Tau = 110		1.07	(0.90, 1.26)		1.06	(0.87, 1.30)
MICS		1.13	(0.99, 1.29)		1.04	(0.91, 1.20)
**Female**	2225					
Complete Cases		1.17	(0.72, 1.90)		0.98	(0.86, 1.13)
IPW		1.15	(0.67, 1.98)		1.00	(0.86, 1.15)
MIRM Tau = 80		1.50	(0.95, 2.35)		1.01	(0.81, 1.26)
MIRM Tau = 110		1.60	(1.00, 2.58)		0.99	(0.76, 1.27)
MICST		1.21	(0.84, 1.74)		1.00	(0.89, 1.14)

**Table 8:** Event time ratio estimates from AFT model for occupational and para-occupational exposure, adjusting for age, sex and race. Highlighted figures indicate statistically significant ( *p* < 0.05) effects

Method	n	Accelerated Failure Time Weibull Model,${\text{exp}}\left({\widehat{\alpha }}_{1}\right)$			
		Occupational Exposure		Para-Occupational Exposure	
		ETR	95% CI	ETR	95% CI
**All**	4507				
Complete Cases		0.98	(0.96, 1.00)	0.99	(0.98, 1.01)
IPW		0.98	(0.96, 1.00)	0.99	(0.98, 1.01)
MIRM Tau = 80		0.98	(0.97, 1.00)	0.99	(0.98, 1.01)
MIRM Tau = 110		0.98	(0.97, 1.00)	1.00	(0.98, 1.01)
MICS		0.98	(0.96, 1.00)	1.00	(0.98, 1.01)
**Black**	560				
Complete Cases		0.92	(0.84, 1.01)	0.98	(0.91, 1.04)
IPW		**0.91**	**(0.84, 0.99)**	**0.94**	**(0.89, 0.996)**
MIRM Tau = 80		0.98	(0.94, 1.02)	**0.95**	**(0.92, 0.97)**
MIRM Tau = 110		0.98	(0.94, 1.02)	**0.95**	**(0.92, 0.98)**
MICS		0.94	(0.88, 1.01)	0.98	(0.93, 1.02)
**White**	3947				
Complete Cases		0.99	(0.96, 1.01)	1.00	(0.98, 1.01)
IPW		0.99	(0.97, 1.01)	1.00	(0.99, 1.01)
MIRM Tau = 80		0.98	(0.97, 1.00)	1.00	(0.99, 1.01)
MIRM Tau = 110		0.98	(0.96, 1.00)	1.00	(0.99, 1.02)
MICS		0.98	(0.96, 1.01)	1.00	(0.98, 1.02)
**Male**	2282				
Complete Cases		0.96	(0.95, 1.00)	0.99	(0.97, 1.01)
IPW		0.98	(0.96, 1.00)	0.99	(0.98, 1.01)
MIRM Tau = 80		0.99	(0.97, 1.01)	0.99	(0.97, 1.01)
MIRM Tau = 110		0.99	(0.97, 1.01)	0.99	(0.97, 1.01)
MICS		0.98	(0.95, 1.00)	0.99	(0.96, 1.02)
**Female**	2225				
Complete Cases		0.97	(0.89, 1.06)	1.00	(0.98, 1.03)
IPW		0.98	(0.91, 1.04)	0.99	(0.98, 1.01)
MIRM Tau = 80		**0.93**	**(0.89, 0.97)**	1.00	(0.98, 1.01)
MIRM Tau = 110		**0.93**	**(0.90, 0.97)**	1.00	(0.98, 1.01)
MICS		0.96	(0.91, 1.03)	1.00	(0.98, 1.02)

When fitting an AFT model, estimates for event time ratios for the effect of occupational or para-occupational exposure only reached statistical significance with MICS and MIRM in the black subpopulation, and with MIRM among female individuals. Overall, this illustrates the benefit of improved efficiency of IPW when accounting for the missing-data mechanism, as observed in simulations.

We assessed the quality of event time imputation using MIRM and MICS in Appendix tables A.5 and A.6. Findings showed that MICS overestimated survival times, while MIRM approaches produced a narrower range of event times. As discussed in the simulation study, this may suggest we failed to capture some unmeasured predictor(s) of life expectancy in the imputation model, though the AFT-based hazard ratio estimates should be minimally biased with this misspecification.

## Discussion

Historical census data linked to administrative records can be a useful resource for epidemiological studies, particularly for associations between exposures and outcomes with historical significance or, as in our use case of asbestos exposure, long incubation periods before population effects can be observed. However, differential success in identifying death records based on individual characteristics can threaten the validity of results. In this paper, we considered the use of historical census data and death records in time-to-event modeling, where death dates may be missing for some individuals. We explored the application of various censoring, weighting, and imputation approaches for handling missing event times, in comparison to a gold-standard approach which assumes that all events that occurred before the date of analysis have been observed. We additionally used weighting to account for the uncertainty associated with equivocal matches.

We show that for estimating log HRs from a Cox PH model, a naïve analysis using only the complete records (weighted (IPW) or unweighted) can lead to biased estimates for the log HR, while censoring on the date of the census can produce unbiased estimates only if the missingness mechanism is independent of the exposure variable of interest, causing severe bias otherwise. Imputing event times based on the conditional survival distribution can be useful for fitting Cox PH models, where point estimates are more robust to the missingness mechanism compared to censoring on the census date. MICS similarly results in the least bias when fitting AFT models, while censoring produces severely biased estimates in all settings. Regarding the precision of the estimates, IPW achieves the greatest efficiency for fitting AFT models (while being minimally biased), while imputation based on conditional survival was most efficient when fitting Cox PH models. Imputation based on conditional survival was also found to be the most accurate among the methods for estimating median survival. MIRM similarly reduced bias when estimating median survival, but the method’s performance was the least consistent, resulting in large bias when linkage quality is covariate-dependent, but minimal bias otherwise. Furthermore, the setting of $\tau$ is not straightforward. $\tau$ set close to the maximum of the distribution led to low bias relative to a smaller $\tau$, but performed poorly when the matching score was dependent on ${Z}_{i}.$ performed better in Cox regression, but higher $\tau$ was preferred for the AFT model. Overall, this investigation illustrates that the strengths and weaknesses of missing data methods may depend on the missingness process as well as the parameters being estimated and models of interest, and such factors should be considered when choosing the methods to address the missing event times. However, MICS most consistently reduced bias across settings in our simulation study.

Differential ascertainment of event times may arise in other study applications involving linked data. For example, a recent study [25] characterized the feasibility of mortality ascertainment using vital status linkage for a diverse historic U.S. pregnancy cohort, finding differences in ascertainment rates by race and across vital record sources. Similar challenges face studies using electronic health record (EHR) data, where the absence of a central, unified health record database leads to variability in the quality and quantity of information that individual EHR sources contain. Thus, obtaining and verifying patient outcomes in cohort studies using EHR data can be subject to differential ascertainment resulting in bias [26]. Ascertainment bias may also be encountered in studies where event records may be less accessible for under-resourced groups, married women with name changes, and those who have switched residences or healthcare.

Our empirical study was not without limitations. Firstly, we assumed the correct specification of survival and imputation models, and that all variables that may impact missingness and/or survival were correctly measured and observed. If missingness is related to variables not collected at the time of the census, or time-varying variables, this may impact our findings, particularly for inverse-probability weights. We also showed the sensitivity of imputation methods to predictor/covariate omission in the imputation model, with AFT model hazard ratios being most robust to misspecification. Furthermore, we did not consider possible interactions between the covariates and the exposure variable in the missingness or survival mechanisms. Robustness of the results to model misspecification should be investigated in future work. In addition, we did not vary the level of missingness and/or censoring in evaluating the performance of our methods. In our data application, we assumed that the observed variables were sufficient in accounting for differential ascertainment.

Finally, we assumed non-informative censoring in our simulations and data application, meaning the censoring mechanism is independent of the time-to-event. However, in practice, this may not hold as life expectancy has increased substantially over the past century with advances in medicine, public health, and nutrition. Administrative record-keeping has also improved over the same time, resulting in greater linkage success for later birth cohorts, who also have longer survival. One way to account for this is by adjusting for calendar effects in survival models.

## Conclusions

Future work should investigate extensions to differential missingness of exposure variables, which may also be found in studies with EHR and genomic data [27, 28], or joint missingness of exposure and outcome variables. The performance of machine-learning approaches, such as random forests and *k*-nearest neighbor algorithms, can also be investigated for this setting. Finally, it should be emphasized that although we have proposed post-hoc measures to account for missing event outcomes, efforts to improve successful data linkages, such as the creation of more centralized databases, or control measures to promote consistency in the quality of data across sources, are preferable.

### Supplementary Information

**Supplementary Material 1.**

## Data Availability

Data summaries generated by the authors are available from the corresponding author upon reasonable request. The original data for the Ambler cohort are not available for redistribution due to privacy concerns.
