# Supplementary material for: Survival analysis under imperfect record linkage using historic census data
Source: BMC Med Res Methodol. 2024 Mar 13;24:67. doi: 10.1186/s12874-024-02194-6 (PMC10935812; doi:10.1186/s12874-024-02194-6)

**Appendix to “Survival analysis under imperfect record linkage using historic census data”**

By Arielle K. Marks-Anglin, Frances K. Barg, Michelle Ross, Douglas J. Wiebe, and Wei-Ting Hwang

**Appendix**

*Figure A.1: Empirical bias and model-based confidence intervals for* $\hat{\beta}_{1}$ *under imputation model misspecification: (1) imputation model excludes* $Z_{i}$*, (2) imputation model includes interaction between* $X_{i}$ *and* $Z_{i}$*. MAR stting with both* $T_{i}$ *and* $R_{i}$ *dependent on* $X_{i}$ *and* $Z_{i}$*.*

*
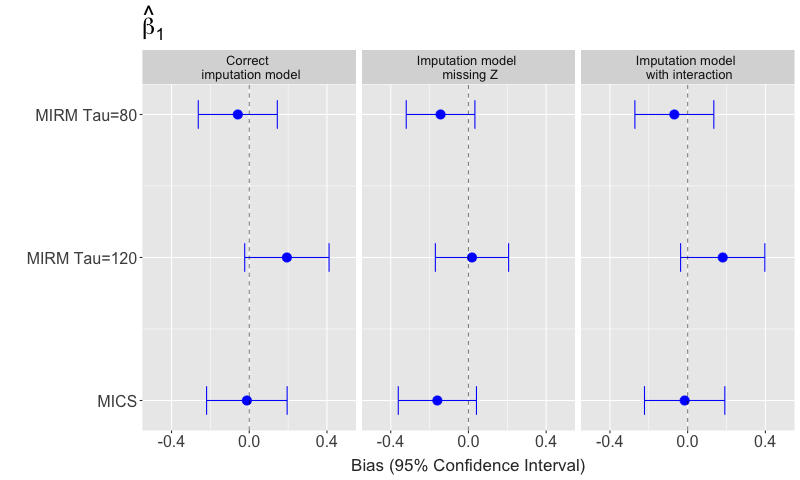
*

*Figure A.2: Empirical bias and model-based confidence intervals for* $\hat{\alpha}_{1}$ *under imputation model misspecification: (1) imputation model excludes* $Z_{i}$*, (2) imputation model includes interaction between* $X_{i}$ *and* $Z_{i}$*. MAR stting with both* $T_{i}$ *and* $R_{i}$ *dependent on* $X_{i}$ *and* $Z_{i}$*.*

*
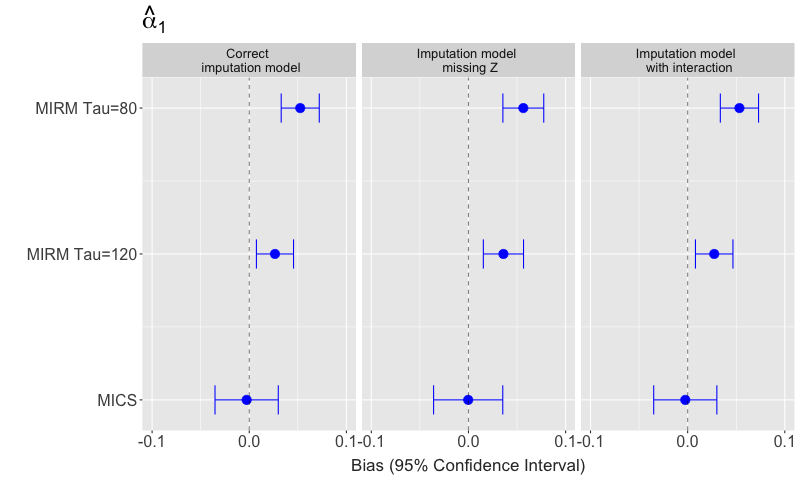
*

*Figure A.3: Empirical bias for* $\hat{M}_{0}$ *and* $\hat{M}_{1}$ *under imputation model misspecification: (1) imputation model excludes* $Z_{i}$*, (2) imputation model includes interaction between* $X_{i}$ *and* $Z_{i}$*. MAR stting with both* $T_{i}$ *and* $R_{i}$ *dependent on* $X_{i}$ *and* $Z_{i}$*.*

*
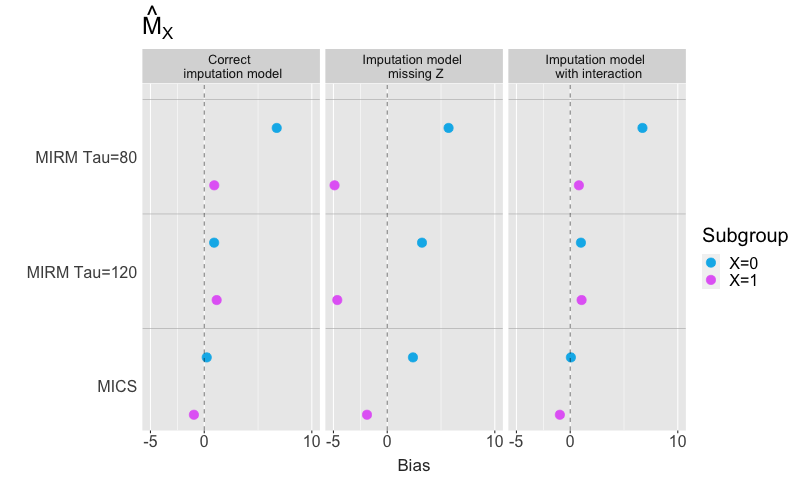
*

*Figure A.4: Empirical bias for* $\hat{M}_{00}$*,* $\hat{M}_{01}$*,* $\hat{M}_{10}$ *and* $\hat{M}_{11}$ *under imputation model misspecification: (1) imputation model excludes* $Z_{i}$*, (2) imputation model includes interaction between* $X_{i}$ *and* $Z_{i}$*. MAR stting with both* $T_{i}$ *and* $R_{i}$ *dependent on* $X_{i}$ *and* $Z_{i}$*.*

*
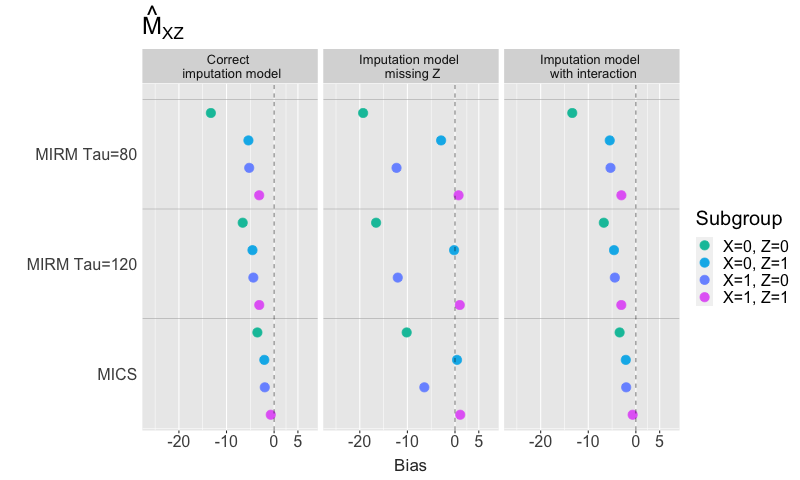
*

*Figure A.5: Histogram of observed and imputed event times for unequivocal matches in Ambler dataset.*

*
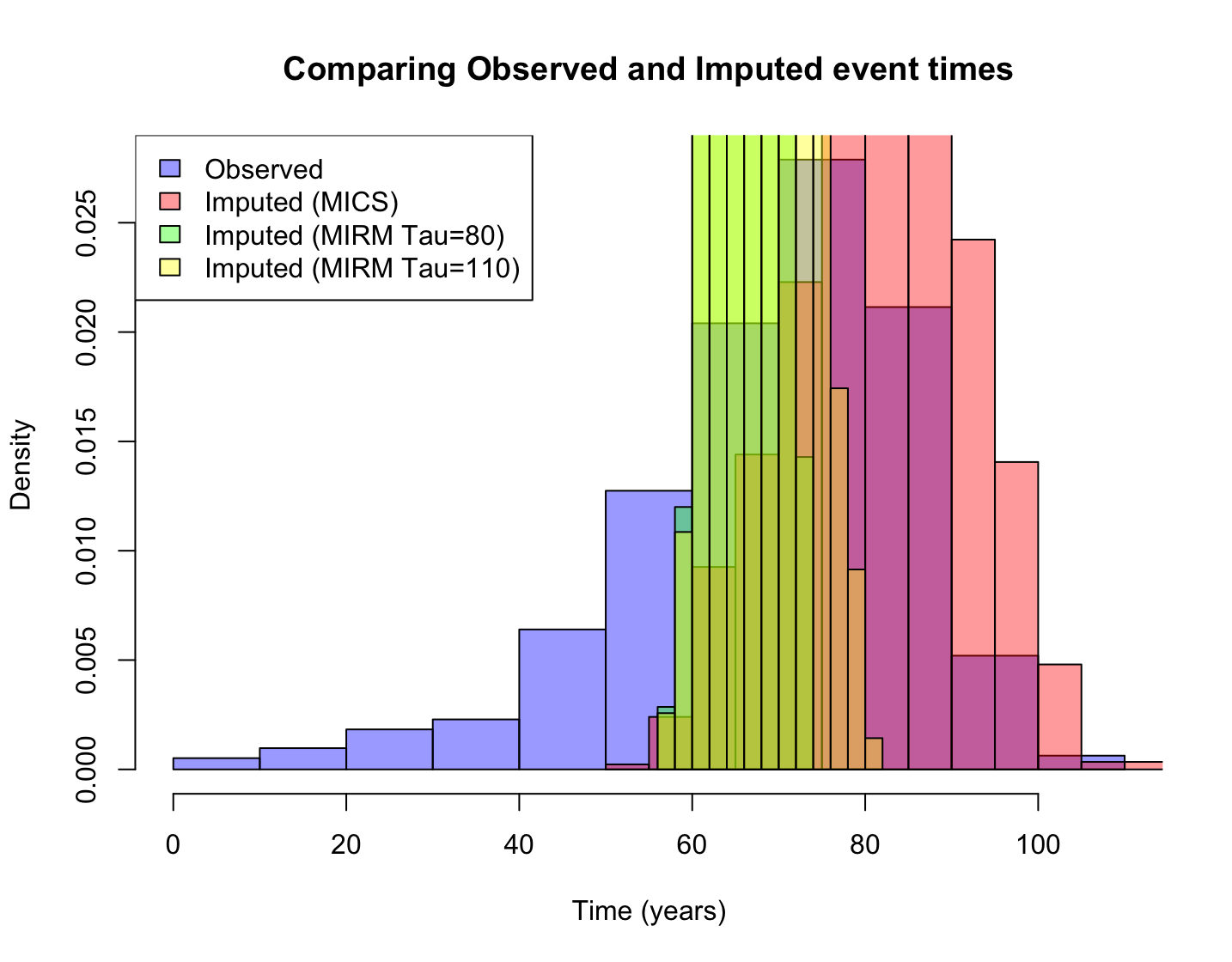
*

*Figure A.6: Kaplan-Meier plot with observed and imputed event times for unequivocal matches in Ambler dataset.*


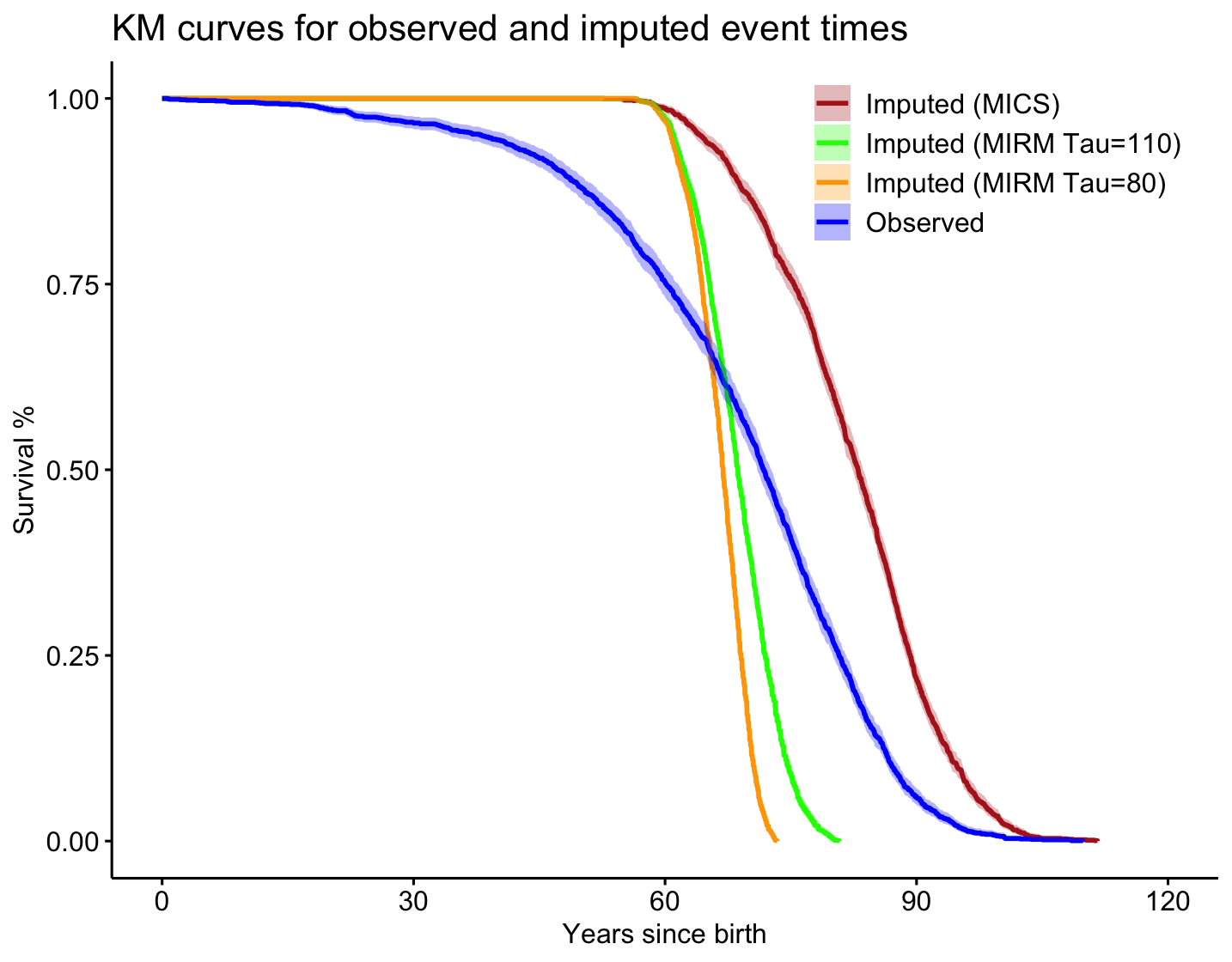

Supplement: Supplementary file 1 — Supplementary Material 1. [file 12874_2024_2194_MOESM1_ESM.docx]
